# Novel Insights Into Genetic Responses for Waterlogging Stress in Two Local Wheat Cultivars in Yangtze River Basin

**DOI:** 10.3389/fgene.2021.681680

**Published:** 2021-05-31

**Authors:** Mingmei Wei, Xiu Li, Rui Yang, Liulong Li, Zhuangzhi Wang, Xiaoyan Wang, Aihua Sha

**Affiliations:** Agricultural College, Yangtze University, Jingzhou, China

**Keywords:** wheat, waterlogging, transcripts, gene regulation, cultivar

## Abstract

Wheat (*Triticum aestivum* L.), the most widely cultivated crop, is affected by waterlogging that limited the wheat production. Given the incompleteness of its genome annotation, PacBio SMRT plus Illumina short-read sequencing strategy provided an efficient approach to investigate the genetic regulation of waterlogging stress in wheat. A total of 947,505 full-length non-chimetric (FLNC) sequences were obtained with two wheat cultivars (XM55 and YM158) with PacBio sequencing. Of these, 5,309 long-non-coding RNAs, 1,574 fusion genes and 739 transcription factors were identified with the FLNC sequences. These full-length transcripts were derived from 49,368 genes, including 47.28% of the genes annotated in IWGSC RefSeq v1.0 and 40.86% genes encoded two or more isoforms, while 27.31% genes in the genome annotation of IWGSC RefSeq v1.0 were multiple-exon genes encoding two or more isoforms. Meanwhile, the individuals with waterlogging treatments (WL) and control group (CK) were selected for Illumina sequencing. Totally, 6,829 differentially expressed genes (DEGs) were detected from four pairwise comparisons. Notably, 942 DEGs were overlapped in the two comparisons (i.e., XM55-WL vs. YM158-WL and YM158-WL vs. YM158-CK). Undoubtedly, the genes involved in photosynthesis were downregulated after waterlogging treatment in two cultivars. Notably, the genes related to steroid biosynthesis, steroid hormone biosynthesis, and downstream plant hormone signal transduction were significantly upregulated after the waterlogging treatment, and the YM158 variety revealed different genetic regulation patterns compared with XM55. The findings provided valuable insights into unveiling regulation mechanisms of waterlogging stress in wheat at anthesis and contributed to molecular selective breeding of new wheat cultivars in future.

## Introduction

Wheat (*Triticum aest*ivum L.), an important source of protein, vitamins, and minerals, contributes about 20% of the calories consumed by humans. It originated from natural hybridization between tetraploid wheat (*T. turgidum* L., AABB genome) and *Aegilops tauschii* Coss (DD genome) (Jia et al., 2013; Zenginbal and Eşitken, 2016; Alaux et al., 2018). It is one of the most widely cultivated crops due to its high yields and nutritional and processing qualities, but its growth and development are restricted by waterlogging (Collaku and Harrison, 2002). The middle and lower reaches of the Yangtze River are the primary region of wheat production in China. In this region, frequent rainfall and excessive irrigation during the rice-growing season can result in water-saturated and compacted soil (Ding et al., 2020a).

Waterlogging is a major abiotic stress in wheat, especially in the middle and lower reaches of Yangtze River basin. The main cause of damage under waterlogging is oxygen deprivation, which affect nutrient and water uptake, so the plants show wilting even when surrounded by excess of water (Malik et al., 2002). Oxygen serves as an electron acceptor in the oxidative phosphorylation pathway. Meanwhile, some biochemical pathways that involve cytochromes, oxidases, and desaturases are also impacted by waterlogging stress (Herzog et al., 2016; Konnerup et al., 2018). Waterlogging has been demonstrated to have some detrimental effects, such as changes in photosynthesis, respiration, and transpiration in the crops, induced senescence of the organs, and a decrease in the accumulation and remobilization of photosynthetic products (Malik et al., 2002; Tan et al., 2008; Ding et al., 2020b). Therefore, many potential key genes and proteins that may play important roles in waterlogging tolerance of wheat were reported in previous studies. For instance, Pan et al. (2019) identified that some proteins related to waterlogging stress, including acid phosphatase, oxidant protective enzyme, and S-adenosylmethionine synthetase 1, may be involved in waterlogging tolerance of wheat. Importantly, in rainfed and irrigated environments, waterlogging may occur at any stage of development because of frequent rainfall (Ding et al., 2020b). The severity of detrimental effects also depend on the growth stage of the plant. In the past, the effects of waterlogging stress on different stages of wheat (e.g., seeds, stem elongation stage, seedling) were also investigated in some studies (Trought and Drew, 1980; Ghobadi et al., 2017; Ploschuk et al., 2020).

Postanthesis is the key period for the formation of wheat grain yield, and waterlogging stress at wheat anthesis seriously impacted wheat yield (Jiang et al., 2004; Tan et al., 2008). Previously, many studies focused on the physiological and biochemical responses on waterlogging stress, as well as genetic regulation for waterlogging tolerance in higher plants (Malik et al., 2002; Zheng et al., 2009; Araki et al., 2012). Some key pathways, such as glycolytic pathway, lignin biosynthesis, and sugar degradation pathways, were reported to involve in the metabolic response to waterlogging stress (Dennis et al., 2000; Li et al., 2014; Nguyen et al., 2016). To our knowledge, few studies about genetic regulation of waterlogging stress at wheat anthesis had been reported. In this study, to find the genetic regulation patterns of wheat at anthesis responding to waterlogging stress, two wheat cultivars that are widely planted in the Yangtze river basin, Xiangmai No. 55 and Yangmai No. 158 (XM55 and YM158), with different tolerance of waterlogging stress were selected as research model. In agricultural practices, YM158 generally exhibits the better performance than XM55 on adapting to the various environment, such as low or high temperature and waterlogging, and XM55 shows high resistance to lodging and some diseases, including wheat scab and powdery mildew. We combined PacBio and Illumina sequencing techniques to find some cues of wheat waterlogging tolerance at anthesis, which is vital to the further germplasm improvement of wheat. The present study contributes to understanding the genetic regulation of waterlogging stress in wheat and provides some clues for the further molecular breeding for waterlogging-tolerant cultivars of wheat.

## Materials and Methods

### Plant Materials and Experimental Design

Two wheat cultivars (i.e., XM55 and YM158), which have been widely planted in the Yangtze River basin, were used in this study. They were sown in the farm of Yangtze University located in Jingzhou, China in growing season. The flag leaves at anthesis of two wheat cultivars were treated with waterlogging for 3 days (WL group), and the wheat at anthesis without waterlogging treatment served as the control group (CK group). Three replicates were performed per treatment for each variety, and the plot areas were 12 m^2^ (2 m × 6 m). At the sowing stage, the base application rate was 90 kg/ha of pure nitrogen from the application of compound fertilizer, and the ratio of available nitrogen N, phosphorus P_2_O_5_, and potassium K_2_O in compound fertilizer was 26:10:15. When 50% plants begin to bloom, in which the plants height is more than 80 cm, the plots were submerged in 2-cm-depth water as waterlogging treatment.

### RNA Extraction and Library Construction for RNA-Seq

Total RNA samples were isolated from the flag leaves of wheat using a commercial Kit (Takara, Dalian, China). Three biological replicates were prepared for each group. The purified RNA was dissolved into 500 ng/μl using RNase-free water, with genomic DNA contamination removed using TURBO DNase I (Promega, Beijing, China). The integrity of the RNA thus prepared was determined with the Agilent 2100 Bioanalyzer (Agilent Technologies, Palo Alto, CA). Only the total RNA samples with RNA integrity number (RIN) ≥ 8 were used for constructing the complementary DNA (cDNA) libraries in PacBio or HiSeq sequencing.

For PacBio sequencing, six samples of XM55 and YM158 were equally mixed, respectively. Finally, two mixed RNA samples were used to construct four libraries and then sequenced using the PacBio RS II platform. Total RNA was reverse transcribed into cDNA using the SMRTer PCR cDNA Synthesis Kit (Takara, Dalian, China), followed by size fractionation using the BluePippin^TM^ Size Selection System (Sage Science, Beverly, MA). The double-stranded cDNA was size selected into 0–5 kb and 4.5–10 kb fractions. Each single molecule real-time (SMRT) bell library was constructed using 1–2 μg size-selected cDNA with the Pacific Biosciences DNA Template Prep Kit 2.0. The binding of SMRT bell templates to polymerases was conducted using the DNA/Polymerase Binding Kit P4 and v3 primers. Sequencing was carried out on the Pacific Bioscience sequel platform (Pacific Biosciences, CA, United States) using C3 reagents with 120-min movies. For Illumina sequencing, the cDNA library was constructed following the manufacturer’s instructions of NEB Next Ultra RNA Library Prep Kit for Illumina. A total of 12 samples were prepared for library construction, each group (i.e., XM55-WL, XM55-CK, YM158-WL, and YM158-CK) had three replicates. The constructed cDNA libraries were sequenced using an Illumina HiSeq X10 platform.

### Subreads Processing and Error Correction of PacBio SMRT Reads

Effective subreads were obtained using the P_Fetch and P_Filter function in the SMRT Analysis Software v2.3 Suite^1^ with default parameters. Circular consensus reads (CCS) was obtained with the P_CCS module. After examining for poly(A) signal, 5′ and 3′ adaptors, only the CCS with all three signals was considered a full-length non-chimetric (FLNC) read. The FLNC sequences were obtained using ToFU pipeline (Gordon et al., 2015). To improve consensus accuracy, we used an isoform-level clustering algorithm, iterative clustering for error correction (ICE), and polished FL consensus sequences from ICE using Quiver. Additional nucleotide errors in FLNC reads were corrected using the Illumina RNA-seq data with the Proovread software (Hackl et al., 2014). The untrimmed sequence was regarded as the result of error correction.

### Discovery of Gene, Isoforms, and Alternative Splicing

After sequence error correction and alignment position correction, FLNC sequences were mapped to the reference genome using Genomic Mapping and Alignment Program (GMAP) with default parameters (Wu and Watanabe, 2005). Subsequently, loci and transcript isoforms were identified according to alignment position of each transcripts. Two alignments of transcripts in the same direction, which overlapped between the initial sites up to 20% and at least one overlapping exon to more than 20%, were identified as the same loci transcript. The new genes and isoforms were detected by comparing loci and isoforms of the transcripts with the loci and isoforms of reference genome annotation. Alternative splicing (AS) events in isoforms constructed from full-length transcripts were classified using ASprofile software (Florea et al., 2013). Using the existing classification and rules in the PlnTFDB database (Riaño-Pachón et al., 2007), the transcription factors were identified by hmmscan program.

### Gene Expression Patterns After Waterlogging Treatment

The Illumina short reads were used to calculate gene expression levels. The raw data was filtered with fastp software (Chen et al., 2018). The clean reads were then mapped to the full-length transcripts using Bowtie2 with default parameters. HTSeq-count program (Anders et al., 2015) was used to determine the reads mapped to individual genes. The gene expression level was determined by fragments per kilobase per million bases (FPKM). DESeq2 (Love et al., 2014) was used to identify the differentially expressed genes with the criterion of fold change ≥ 2 and FDR < 0.01. The gene ontology (GO) and Kyoto Encyclopedia of Genes and Genomes (KEGG) pathway enrichments were performed with the Database for Annotation, Visualization, and Integrated Discovery (DAVID) online tool.

### Validation of RNA-Seq Data by RT-qPCR

Six RNA samples, including waterlogging and control groups in XM55 cultivar, were used for quantitative reverse transcription PCR (qRT-PCR). First-strand cDNA was prepared from 5 μg total RNA using the SuperScript First-Strand cDNA Synthesis Kit (Invitrogen, United States). The reactions were performed on an ABI PRISM 7300 System (Applied Biosystems, United States) following the manufacturer’s instructions. Each reaction mixture was 20 μl, containing 10 μl of SYBR Premix Ex Taq (Takara, Japan). The reactions for each gene were conducted in triplicate with the thermal cycling conditions as follows: 95°C for 15 min, followed by 40 cycles of 95°C for 10 s, 57°C for 20 s, and 72°C for 30 s; the last stage is 95°C for 10 min, 57°C for 5 s, and 95°C. The primer specificity was confirmed by melting curve analysis. The relative expression levels of the tested genes were calculated using the 2^–ΔΔCt^ method with normalization to that of the reference genes.

## Results

### Identification of Wheat Full-Length Transcripts

**FIGURE 1:**
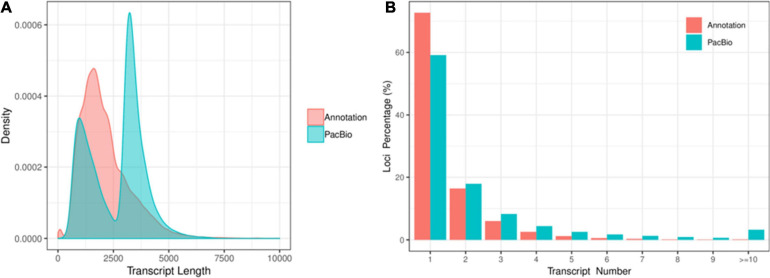
**(A)** The distribution of transcript length and the **(B)** isoform number between the full-length transcripts and the transcripts in IWGSC RefSeq v1.0 annotation.

A total of 701,747 and 587,454 CCS reads were generated for XM55 and YM158 cultivars, respectively (Supplementary Table 1). Of these, 947,505 high-quality FLNC reads were obtained, 85.47% of which were mapped to the reference genome IWGSC RefSeq v1.0 (Supplementary Table 2). The mapping results are available in figshare^2^. A total of 117,546 FLNC reads were uniquely mapped to the reference genome. The average length of the non-redundant transcripts was 3,294 bp, which was much higher than that of transcripts annotated with genome (1,295 bp) (Figure 1A and Supplementary Table 3). These transcripts were derived from 49,368 genes, including 47.28% (49,368/104,415) of the genes annotated in IWGSC RefSeq v1.0^3^. Of these genes, 40.86% (20,171/49,368) genes were multiple-exon genes, encoding two or more isoforms, while 27.31% (28,514/104,415) genes in IWGSC RefSeq v1.0 were multiple-exon genes encoding two or more isoforms (Figure 1B). These results indicated that PacBio SMRT sequencing method could significantly improve the genome annotation of wheat in terms of the length and the quantity of transcripts.

### Characterization of Novel Genes and Isoforms

We identified a total of 11,099 (22.48%) novel genes by comparing with the previous genome annotation of IWGSC RefSeq v1.0. These novel genes encode 23,498 isoforms. Meanwhile, 67,151 novel isoforms were detected from the 25,273 genes annotated in the IWGSC RefSeq v1.0. We integrated and merged these PacBio data with the genome annotation, and the gene models of wheat genome were renewed, which are shown in a GFF file^4^. Furthermore, we searched the isoforms against the public protein databases, and the results showed that 79.13% isoforms identified from full-length transcripts had homologous hits in the databases, as well as the isoforms identified with the IWGSC RefSeq v1.0 annotation (Table 1). Furthermore, 692 unannotated novel isoforms (20.69%) from novel genes in this study and 4,617 (3.08%) isoforms from genome annotated genes were characterized as long non-coding RNAs (lncRNAs), respectively.

**TABLE 1:** The transcript annotation of the novel discovered transcripts with the public databases.

**Database**	**PacBio data**	**Ratio**	**Genome data**	**Ratio**
Total transcripts	23,498		243,594	
NR	18,593	79.13%	236,047	96.90%
GO	8,245	35.09%	160,965	66.08%
KO	5,324	22.66%	86,916	35.68%
KOG	3,112	13.24%	78,468	32.21%
Swiss-Prot	3,967	16.88%	85,358	35.04%
Unannotated	4,861 (692 lncRNAs)	20.69%	7,494 (4617 lncRNAs)	3.08%

*NR indicates NCBI non-redundant protein sequence database; GO indicates gene ontology database; KO represents KEGG Orthology database; KOG represents the database of clusters of orthologous groups for eukaryotic complete genomes; Swiss-Prot indicates SWISS-PROT protein sequence data bank.*

### Alternative Splicing, Fusion Genes, and lncRNA in Wheat

The AS events were detected in the XM55 and YM158 with waterlogging and control groups (XM55-T, XM55-CK, YM158-T, YM158-CK). The AS events were classified into 6 major types and 10 categories [(X)SKIP, (X)MSKIP, (X)IR, (X)MIR, and (X)AE]. The summary of AS events is shown in Supplementary Table 4. Likewise, we identified 1,542 full-length transcripts that map into two or more loci in the genome. These fusion transcripts corresponded to 209 chimeric genes (Supplementary Table 5). Among these fusion genes, the fusion gene 15 consisted of 83 fusion transcripts, which contained NC_030641.1 and NW_016115358.1. To identify lncRNAs, the coding potential of these novel genes that did not have any homologous sequence in public databases was evaluated using Coding Potential Assessing Tool (CPAT). Finally, we obtained a total of 5,309 lncRNAs by using CPAT software (Supplementary Table 6). The length of lncRNAs varied from 209 to 6,605 bp, with a length ≥ 1,000 bp accounted for 70.37%.

### Gene Expression Profiling of XM55 and YM158 With Waterlogging Stress

To assess gene expression dynamics of wheat under waterlogging stress, the Illumina short reads were obtained for the determination of gene expression levels. A total of 425,726,446 clean reads were yielded for the Illumina libraries (Supplementary Table 7). To evaluate the reliability of the three biological replicates, correlation analysis was performed based on the FPKM values. The coefficients ranged from 0.9423 to 0.9783, which indicating that our RNA-seq data was reliable (Supplementary Figure 1). After mapping with the full-length transcripts, a total of 91,923 genes were detected. Of these, 85,443 (92.95%), 83,314 (90.63%), 83,437 (90.77%), and 82,964 (90.25%) genes were expressed in the XM55-WL, XM55-CK, YM158-WL, and YM18-CK, respectively. Notably, some genes, including ribulose-1,5-bisphosphate carboxylase/oxygenase small subunit, metallothionein-like protein 1, and photosystem I subunit VII, were highly expressed in these samples (FPKM > 1,000). Some genes, such as BLT101, wheat cold induced 16, abscisic stress-ripening protein 2, CCD-A4, thioredoxin H-type 4, cell division protease ftsH-like protein, chloroplastic, serine-glyoxylate aminotransferase, crystal structure of wheat cyclophilin A, and CCD-D1 were only highly expressed in YM158 with waterlogging stress.

### Differentially Expressed Genes in Response to Waterlogging Stress in XM55 and YM158

**FIGURE 2:**
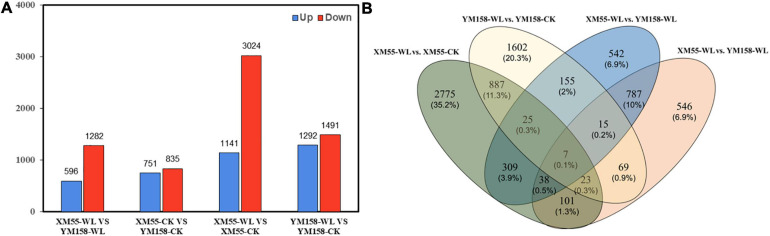
**(A)** The number of differentially expressed genes (DEGs) identified from four pairwise comparisons and **(B)** the overlap of the DEGs among four pairwise comparisons.

**FIGURE 3:**
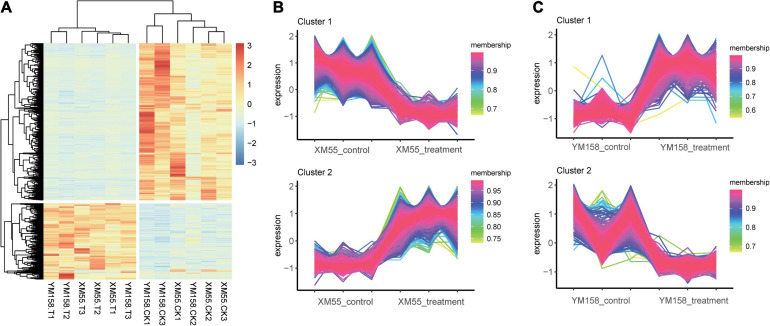
**(A)** Clustering analysis of the overlapping differentially expressed genes (DEGs) and the expression trends in the treatment and control groups in **(B)** XM55 and **(C)** YM158.

Totally, 4,165 DEGs (1,141 and 3,024 up-/downregulated) were identified between waterlogging and control groups in XM55. Likewise, 3,783 DEGs were detected between waterlogging and control groups in YM158 (Figure 2A). All DEGs are listed in Supplementary Table 8. Importantly, 942 DEGs were overlapped in both two pairwise comparisons (Figure 2B), inferring that these genes play important roles in waterlogging tolerance. The expression levels of these DEGs could be grouped into two clusters (Figure 3A), and most of them (653 genes) were downregulated in the waterlogging groups (Figures 3B,C). Based on the annotation with KEGG database, these DEGs were involved in the mitogen-activated protein kinase (MAPK) signaling pathway, plant hormone signal transduction, and starch and sucrose metabolism. Meanwhile, the DEGs between XM55 and YM158 were also identified. We obtained 1,586 and 1,878 DEGs by the pairwise comparisons of XM55-CK vs. YM158-CK and XM55-WL vs. YM158-WL, respectively (Figure 2A). Notably, 1,282 genes of 1,878 DEGs (e.g., *ATPF1A*, *psbA*, *psaB*, *psaH*, *petF*, *psbR*, *LHCB1*) were significantly upregulated in YM158-WL compared with XM55-WL, and these genes were significantly enriched in two key pathways, including photosynthesis (ko00195) and photosynthesis-antenna proteins (ko00196). It could be inferred that the photosynthesis play key roles in waterlogging resistance, and those candidate genes involved in photosynthesis may account for the different genetic regulation mechanisms of waterlogging stress in two cultivars, which have different capacity for waterlogging resistance.

**FIGURE 4:**
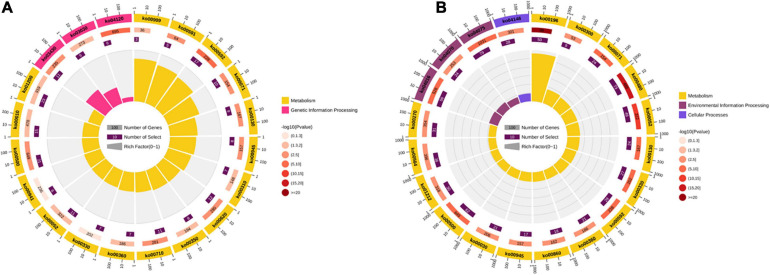
The Kyoto Encyclopedia of Genes and Genomes (KEGG) pathway enriched in the comparisons of **(A)** XM55-WL vs. YM158-WL and **(B)** XM55-CK vs. XM55-WL.

GO enrichment analyses were performed with all the DEGs. The results showed that most DEGs were enriched categories and comprised metabolic, cellular, and single-organism processes. In the category of biological process, the DEGs in both comparisons were associated with several important processes involving in stress tolerance, such as activation of GTPase activity, hormone transport, hyperosmotic response, phospholipid metabolic process, phosphorus metabolic process, phosphorylation, regulation of proteolysis, regulation of protein ephosphorylation, response to cold, and response to osmotic stress. In the category of molecular function, the items of fatty acid transporter activity, FMN binding, and oxidoreductase activity were detected in both four comparisons. The KEGG pathway enrichment analyses were carried out in the comparisons of XM55-WL vs. YM158-WL and XM55-CK vs. XM55-WL. Totally, 17 and 39 pathways were significantly enriched in the two pairwise comparisons, respectively (Figure 4 and Supplementary Table 9). Some pathways, including carbon fixation in photosynthetic organisms, DNA replication, lysine degradation, mismatch repair, pyruvate metabolism, sesquiterpenoid and triterpenoid biosynthesis, and tyrosine metabolism were only detected in XM55-WL vs. YM158-WL. Additionally, some pathways, such as fatty acid biosynthesis and metabolism, MAPK signaling pathway, peroxisome, arachidonic acid metabolism, galactose metabolism, and plant hormone signal transduction, were enriched in XM55-WL vs. XM55-CK.

### Waterlogging Stress Affected Photosynthesis and Steroid Hormone Biosynthesis in Wheat

With waterlogging treatment, a total of 96 genes related to photosynthesis and photosynthesis-antenna proteins were significantly downregulated in wheat. In this study, the photosynthesis (ko00195) and photosynthesis-antenna proteins (ko00196) were significantly enriched with the DEGs between waterlogging and control group in both two cultivars. The expression levels of photosynthesis-related genes, such as *PsbQ*, *PsbO*, and *petF*, were significantly lower in waterlogging groups than those in control groups. Meanwhile, some key genes involved in light-harvesting chlorophyll protein complex, e.g., *LHCB1*, *LHCB3*, *LHCB5*, *LHCA1*, and *LHCA4*, were also downregulated after waterlogging treatment.

Meanwhile, we found that the pathway of steroid biosynthesis was enriched in the waterlogging treatment groups, and the genes in this pathway (e.g., *CYP51G1*, *CYP710A*, *CYP734A1*, *TGL4*, and *LIPA*) were downregulated in the waterlogging treatment groups compared with control groups in XM55 variety. Interestingly, these key genes (e.g., *CYP51*, *CYP85A2*, *TGL4*, *SMO1*, and *CYP734A1*) were upregulated in YM158 after waterlogging treatment. In this case, it revealed the genetic superiority of YM158 on waterlogging tolerance by upregulation of steroid biosynthesis. Moreover, some key genes in the downstream pathway, brassinosteroid biosynthesis, were upregulated with waterlogging stress in both two cultivars, which promoted the biosynthesis of 26-hydroxy-brassinolide and 26-hydroxy-castasterone. Brassinosteroids are a group of plant steroid hormones, which can mediate the downstream hormone signal transduction. In this study, we found that several genes (e.g., *BZR1/2*, *BSK*) involved in plant hormone signal transduction were upregulated in wheat with waterlogging stress. We speculated that the upregulation of upstream pathways of steroid and brassinosteroid biosynthesis promoted the inactivation of BIN2, which furthermore regulated the dephosphorylation and activation of BZR1 and BZR2 (also known as BES1). Additionally, we found that waterlogging stress could induce the upregulation of *BZR1* and *BES1*, and the increased *BZR1* and *BES1* expression levels could promote the derepression of BR biosynthesis.

### Validation of RNA-Seq Data With qPCR

**FIGURE 5:**
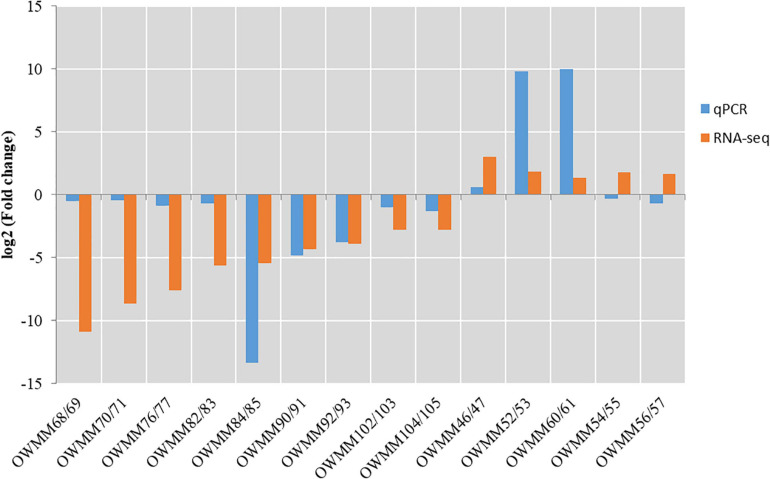
The validation of RNA-seq data using quantitative PCR (qPCR) method.

To verify the DEGs identified by pairwise comparison, the expression levels of 14 DEGs, including 5 upregulated and 9 downregulated genes in XM55-WL vs. YM158-WL were evaluated using RT-qPCR method. The primer sequences are shown in Supplementary Table 10. The expression levels of the selected genes except OWMM54/55 and OWMM56/57 detected with RT-qPCR method were consistent with the expression levels calculated from RNA-Seq data (Figure 5), indicating that the RNA-seq data in this study was reliable.

## Discussion

In the middle and lower reaches of Yangtze river basin, the plant area of winter wheat accounts for about 12% of the whole plant area of China, but frequent waterlogging severely impaired the wheat production during the growing season. Many studies have reported that waterlogging significantly decreases grain yield due to an inhibition of photosynthesis, respiration, and transpiration, resulting in an accumulation and remobilization of assimilate in vegetative organs and yield components (Tan et al., 2008; Araki et al., 2012; Arguello et al., 2016; Ding et al., 2020b). However, those investigations were conducted with different wheat cultivars and various growing environments. Therefore, it is quite urgent to evaluate the effects of waterlogging on wheat growth of local wheat cultivars. In the present study, XM55 and YM158 were selected due the fact that these two cultivars have been widely planted in the middle and lower reaches of Yangtze River basin in China. In addition, in agriculture practices, these two cultivars showed different tolerance ability on waterlogging stress, and YM158 has superiority of waterlogging resistance. Some studies attempted to find the key stage (Romina et al., 2014), such as stem elongation (Shao et al., 2013), anthesis (de San Celedonio et al., 2014), and grain filling (Ploschuk et al., 2018), on waterlogging in wheat, but different conclusions were reported. We speculated that the different environments and cultivars may affect the research conclusions.

Due to the polyploidy of wheat, improving the completeness of genome assembly is still a great challenge for bioinformatics scientists. Hence, we used PacBio sequencing method to detect the transcripts in genome. After annotation of those transcripts, we found that the numbers of genes and isoforms detected in this study are more than those from genome annotation. Meanwhile, we also obtained many fusion genes and identified AS events. These results contributed to filling the gaps of genome annotation for wheat. With those high-quality full-length transcripts, we calculated the gene expression levels of wheat leaves in waterlogging treatment and control groups. First, we found that the photosynthesis of wheat was adversely affected by waterlogging stress, and many genes involved in photosynthesis were downregulated in waterlogging groups for two cultivars. Previously, many studies (Pang et al., 2004; Tiryakioğlu et al., 2015) have reported that, at the physiological level, waterlogging caused reduced photosynthetic rate, chlorophyll content, transpiration, average leaf water-use efficiency, etc. in wheat. At genetic levels, the downregulation of the photosynthesis-related genes coincided with the previous studies (Shao et al., 2013; Gao et al., 2021). Besides, several studies have demonstrated that some quantitative trait loci (QTLs) were associated with waterlogging tolerant in wheat (Yu and Chen, 2013; Ballesteros et al., 2015). For instance, Ballesteros et al. (2015) identified two QTL regions on chromosome 6D that were associated with root biomass and chlorophyll content. However, the key genes identified from the QTL regions have not been reported in previous studies, and further studies should be performed in the future, which will be conducive to the molecular breeding of waterlogging-tolerant wheat cultivars.

Plant steroid hormones play major roles in abiotic stress tolerance in plants. Brassinosteroids (BRs), a group of steroid hormones, show quicker response at very low concentrations (Kanwar et al., 2012), and it generally occurs in all parts of plants including roots. Beside their roles in plant growth and development, BRs also mediate responses to stresses such as heat, cold, and drought stress (Nolan et al., 2020). In this study, *BSK* and *BZR1/2* were upregulated in wheat with waterlogging stress. Previous studies (He et al., 2002; Eremina et al., 2016; Ye et al., 2019) have demonstrated that *BZR1*, the central transcription factor in BR signaling, *BIN2*, and *CESTA* positively regulates cold responses by mediating activation of cold-responsive genes in *Arabidopsis*. Given that BRs are conserved in plant species, we could infer that the *BZR1/2* also regulates the short-term waterlogging stress (hypoxia stress) by mediating the BRs signaling and biosynthesis. Meanwhile, we found that YM158 and XM55 showed difference in gene expression patterns in the upstream of BRs biosynthesis, which may account for the sensitivity of waterlogging stress. More efforts should focus on untangling the roles of the specific BR signaling components in stress responses and revealing their spatiotemporal regulation in the future study.

## Conclusion

In summary, this study integrating PacBio and Illumina sequencing methods aimed to find the genetic regulation of waterlogging stress in two local wheat cultivars (XM55 and YM158). With the full-length transcripts, we obtained many fusion genes and detected many AS events. Importantly, the gene expression patterns of waterlogging and control groups were accessed in two cultivars, and some key DEGs and pathways related to the waterlogging response, including steroid metabolism and biosynthesis and its downstream BRs biosynthesis and hormone signal transduction, were identified. This study contributed to understanding the genetic regulation of wheat waterlogging stress and providing valuable data for future selective breeding of new wheat cultivars.

## Data Availability Statement

The raw data of Illumina short reads and Pacbio long reads has been deposited into NCBI SRA database with accessions of SRR13264476-SRR13264489.

## Author Contributions

XW and AS designed the experiments. MW and XL conducted data analysis and wrote the manuscript. LL conducted waterlogging treatment. RY conducted qRT-PCR. ZW conducted data analysis. All authors contributed to the article and approved the submitted version.

## Conflict of Interest

The authors declare that the research was conducted in the absence of any commercial or financial relationships that could be construed as a potential conflict of interest.

## Footnotes

1 http://www.pacb.com/devnet/

2 https://doi.org/10.6084/m9.figshare.14403905.v1

3 https://wheat-urgi.versailles.inra.fr/Seq-Repository/Annotations

4 https://doi.org/10.6084/m9.figshare.14403896.v1

## Supplementary Material

Supplementary Figure 1 Correlation analysis of the FPKM (fragments per kilobase per million mapped reads) values of all samples.

Supplementary Table 1 Summary of the reads produced by PacBio sequencing.

Supplementary Table 2 Summary of the mapping ratios of FLNC reads to reference genome.

Supplementary Table 3 The gene and isoform annotations IWGSC RefSeq v1.0 and PacBio data.

Supplementary Table 4 Statistics of alternative splicing events.

Supplementary Table 5 List of fusion genes identified with the full-length transcripts of wheat.

Supplementary Table 6 | Identification of LncRNAs with the full-length transcripts of wheat.

Supplementary Table 7 Reads produced from 12 samples by Illumina sequencing.

Supplementary Table 8 The list of Known DEGs. “1” represents the gene is differentially expressed gene (DEG) in the comparisons.

Supplementary Table 9 The enrichment of KEGG pathways with DEGs of flag leaves in wheat (*P* < 0.05).

Supplementary Table 10 The primers for qRT-PCR analyses.
