# Supplementary material for: Novel Insights Into Genetic Responses for Waterlogging Stress in Two Local Wheat Cultivars in Yangtze River Basin
Source: Front Genet. 2021 May 31;12:681680. doi: 10.3389/fgene.2021.681680 (PMC8201782; doi:10.3389/fgene.2021.681680)

**Novel insights into genetic responses for waterlogging stress in two local wheat cultivars in Yangtze River basin**

Mingmei Wei^#^, Xiu Li^#^, Rui Yang, Liulong Li, Zhuangzhi Wang, Xiaoyan Wang***,** Aihua Sha*

Agricultural College, Yangtze University, Jingzhou 434025, China

Fig. S1. Correlation analysis of the FPKM (fragmentss per kilobase per million mapped reads) values of all samples. The average coefficient for the three replicates at the XM55-WL(A), XM55-CK(B), YM158-WL(C) and YM158-CK(D) was 0.9783, 0.9537, 0.9423 and 0.9720, respectively.


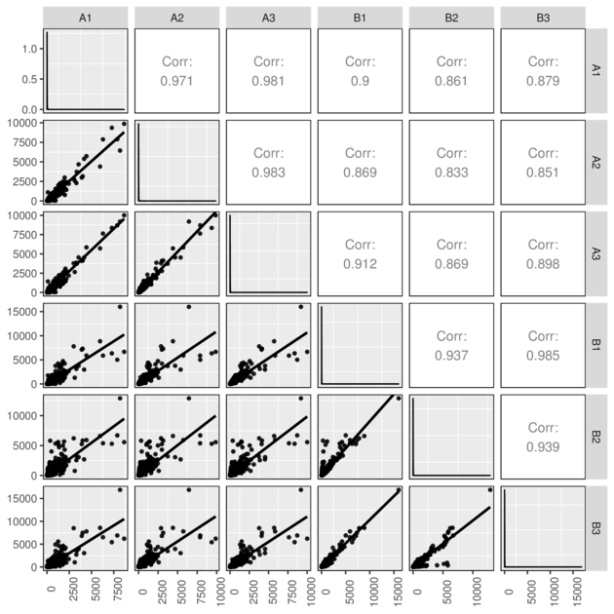

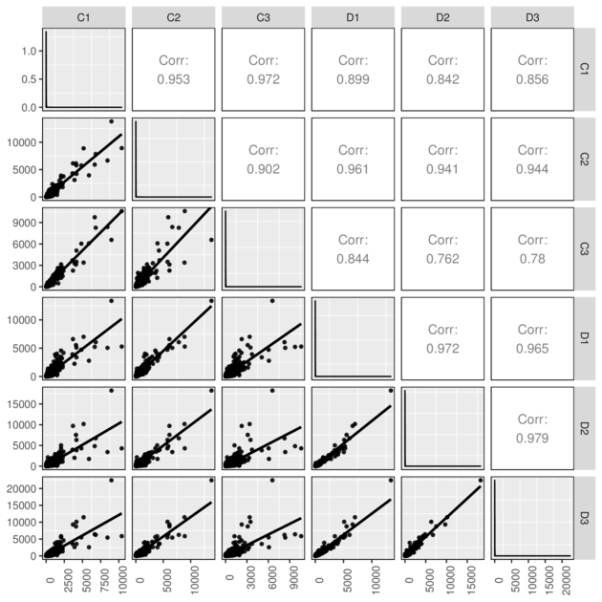

Supplement: Supplementary Figure 1 — Correlation analysis of the FPKM (fragments per kilobase per million mapped reads) values of all samples. [file Data_Sheet_1.docx]
